# Recommendations for Successful Implementation of the Use of Vocal Biomarkers for Remote Monitoring of COVID-19 and Long COVID in Clinical Practice and Research

**DOI:** 10.2196/40655

**Published:** 2022-11-15

**Authors:** Aurelie Fischer, Abir Elbeji, Gloria Aguayo, Guy Fagherazzi

**Affiliations:** 1 Deep Digital Phenotyping Research Unit Department of Precision Health Luxembourg Institute of Health Strassen Luxembourg

**Keywords:** vocal biomarker, COVID-19 symptoms, digital health, remote monitoring, artificial intelligence, voice, COVID-19, Long COVID, digital health solution, voice-based technology, health technology, health monitoring, digital health monitoring, health care application, remote patient monitoring

## Abstract

The COVID-19 pandemic accelerated the use of remote patient monitoring in clinical practice or research for safety and emergency reasons, justifying the need for innovative digital health solutions to monitor key parameters or symptoms related to COVID-19 or Long COVID. The use of voice-based technologies, and in particular vocal biomarkers, is a promising approach, voice being a rich, easy-to-collect medium with numerous potential applications for health care, from diagnosis to monitoring. In this viewpoint, we provide an overview of the potential benefits and limitations of using voice to monitor COVID-19, Long COVID, and related symptoms. We then describe an optimal pipeline to bring a vocal biomarker candidate from research to clinical practice and discuss recommendations to achieve such a clinical implementation successfully.

## Introduction

### Management of COVID-19 and Long COVID

Since February 2020, the COVID-19 pandemic has mobilized the entire research, medical, and pharmaceutical community for treatment and vaccine development as well as care and disease management of patients with COVID-19. Many countries adopted strategic measures to fight the pandemic, such as lockdowns, social distancing, or contact tracing, to reduce transmission [1]. In parallel, we observed rapid a development of digital tools to support the health care system and an increased use of telemedicine and teleconsultations. COVID-19 infection is caused by the SARS-CoV-2 virus and can take several forms, from asymptomatic to moderate or severe illness. At the onset of the illness, common symptoms are fever, cough, dyspnea, myalgia fatigue, loss of taste or smell, and sometimes gastrointestinal symptoms. Complications include acute respiratory distress syndrome, anemia, or acute cardiac injury [2].

After the acute phase, it has been estimated that up to 68% of patients with COVID-19 will have persisting symptoms after 6 months, and 49% after a year [3]. Long COVID syndrome is defined as a set of symptoms persisting and fluctuating beyond 4 weeks after infection, leading to long-term support and care needs [4]. Long COVID is multiorgan and can affect lungs, heart, brain, kidney, and blood vessels. Long COVID also affects cognitive functions and mental health with diverse symptoms such as brain fog [5], confusion, memory deficiencies, and even posttraumatic stress disorder [6]. The most frequently reported symptoms are fatigue, abnormalities of smell and taste, anxiety, sleeplessness, and dyspnea [4]. The severity of the acute illness seems not to be directly related to the development of Long COVID, and many people with Long COVID did not return to the same level of work and quality of life as before COVID-19 infection [7]. The absence of treatment and the specificities of Long COVID in terms of symptom types and fluctuation over time justifies the need to develop solutions for objective and qualitative symptom monitoring rapidly. As such, a panel of experts from the National Institute for Health and Care Excellence recently recommended developing telemonitoring and encouraging self-management of acute and Long COVID symptoms in a tailored and accessible way for each patient [8]. In particular, at-home self-monitoring is encouraged [9].

### From Traditional to Enhanced Approaches of Patient Monitoring

The medical care of patients in real-life situations has been rapidly evolving since the pandemic started. Video or phone consultations have largely replaced traditional visits to the family doctor or specialist and have shown the utility [10] of these formats to follow up patients who are not able to travel or are located in geographic regions lacking medical doctors. In parallel, the use of medical devices for self-monitoring of physiological parameters such as blood pressure or blood glucose level has also increased. The next development step would be to unify and standardize telemedicine solutions with enhanced teleconsultations, including a real-time assessment of key physiological parameters. This would optimize the consultation time and enable a personalized follow-up with increased communication between the health care professionals and the patients.

Regarding clinical research, there was already, before the pandemic, a global trend toward completely remote, decentralized clinical trials [11,12]. The pandemic has now accelerated such digitization. Participant monitoring in clinical trials is progressively moving away from a paper-based approach (ie, paper informed consent, questionnaires, and medical records) to fully remote or hybrid setups, where patients’ visits on-site and remote, ‘in-between visits,’ and follow-up in real-life are combined. Digitization and decentralization of clinical trials encompass three axes: digital participant recruitment and retention, digital data collection, and digital analytics [13], based on electronic documents (eg, e-consent and electronic case report forms), virtual study visits, and physical self-measurements. Digital data collection facilitates and standardizes data quality, whereas digital analytics using artificial intelligence techniques like machine and deep learning methods allow for deep phenotyping of participants.

### Digital Technologies for the Clinical Management of Patients With COVID-19

Technologies already exist to facilitate remote patient monitoring in clinical practice or research, such as electronic patient-reported outcomes, sensors or devices to measure physiological parameters at home (eg, Holter for electrocardiograms, blood pressure, and heart rate), video-based methods, or mobile phone–based remote symptom monitoring systems [14]. In the context of COVID-19, hundreds of contact tracing mobile apps and artificial intelligence–based radiological technologies to facilitate early detection of COVID-19 emerged early during the pandemic [15]. In parallel, several digital technologies have been developed to respond to different patient needs, including diagnosis, prevention, treatment, adherence, lifestyle, or patient engagement [16]. For example, a smartwatch application has been developed in Germany to help COVID-19 diagnosis based on a few vital signs [17], and 2 remote monitoring systems—Telecare-COVID (based on phone calls) and CareSimple-COVID (a telemonitoring app)—are used in Canada and are well accepted by the users [18].

Voice assistants have also been identified as an innovative tool for health care services in the context of a pandemic, as a tool for health information exchange, for remote monitoring or to maintain continuous care with teleconsultations [19].

Voice is an easy-to-collect source of information, requiring less time than completing a questionnaire, being noninvasive, and inducing less burden for patients or study participants. The first use of voice dates back to 2003 in clinical studies and health care services with interactive voice response system [20], a phone-based system mainly used for patient randomization. Interactive voice response system was also used for symptom monitoring by calling participants and encouraging them to answer questions on their health status and in return, patients could receive specific recommendations to manage their treatment. However, voice is in itself a rich medium providing information on health status and emotions, allowing for a richer characterization of patients through the use of so-called vocal biomarkers. This opens many perspectives of using voice besides the practical aspect of using it as a collection tool.

As we are now at a turning point in telemedicine, we believe that the use of vocal biomarkers is among the most promising approaches to improve patient monitoring of COVID-19–related symptoms.

In the following sections, we provide an extensive description of the potential benefit and limitations as well as recommendations for the development of a digital health solution based on vocal biomarkers. Since the pandemic revealed specific needs, these recommendations are elaborated in the COVID-19 context but can be easily generalized to other diseases or symptoms.

## Using Vocal Biomarkers for Remote Symptom Monitoring in the Future

### What is a Vocal Biomarker?

As mentioned before, voice is a rich medium, characterized by thousands of different features, potentially affected by our health status. Thus, a vocal biomarker is an extension of a classical biomarker, a factor objectively measured and evaluated representing a biological or pathogenic process or a pharmacological response to a therapeutic intervention [21]. It can also be used as a surrogate marker of a clinical end point. A vocal biomarker can therefore be defined as a signature, a feature, or a combination of features from the audio signal of the voice associated with a clinical outcome. It must have all the properties of a traditional biomarker, needing to be analytically validated and qualified using an evidentiary assessment. A vocal biomarker can be used to monitor patients, diagnose a condition, or grade the severity of a disease [21].

Vocal biomarkers have already been described in pathologies such as Parkinson disease, depression, and cardiovascular diseases with the potential of early diagnosis or disease progression markers [21], but none of them is used in clinical practice yet. Since voice features can be specifically associated with these different pathologies, one can extrapolate that similar voice features could be associated with a COVID-19 infection or with a consequence of COVID-19. COVID-19 infection or complications can affect voice through different mechanisms. For example, respiratory insufficiency can lead to reduced airflow, and therefore, changes in voice parameters [22]. Other studies showed that voice quality was reduced in patients with COVID-19 due to repeated cough, laryngeal or pharyngeal erythema, or sore throat [22-24].

### Potential Benefits and Limitations of Using Vocal Biomarkers in the Context of COVID-19

The first developments based on the use of vocal biomarkers in the context of COVID-19 were meant to enable the detection of COVID-19 infection. Vocal biomarkers for COVID-19 detection in cough and voice have been developed and could one day serve as a screening tool on a very large scale and in a short period of time, for example, at airports or border controls, leading to a direct benefit for the pandemic management [25,26].

Another benefit of vocal biomarkers to monitor COVID-19–related symptoms remotely is a reduced burden for the user by limiting the clinical visits on-site, replacing tedious questionnaires and physical examinations, and facilitating the reporting of symptoms or adverse events [27]. It could also allow for simultaneous monitoring of several COVID-19–related symptoms, with early detection of a worsening in the health or mental condition, or on the other hand, serve as a proxy to assess treatment or rehabilitation effectiveness. All of these benefits combined could in turn lead to reduced risk of hospitalization and increased quality of life.

From a clinician or a researcher’s perspective, the use of vocal biomarkers can facilitate and objectivize patient evaluation, in particular when the results are transmitted by a visualization tool that might be easier to interpret than questionnaires. Vocal biomarkers could also serve as a proxy to assess the benefits of a rehabilitation program for people with Long COVID. As the collection of voice recordings is fast, fun, and limits the burden for patients, it could also reduce attrition in clinical trials. Lastly, voice collection reduces costs and allows for fast and high-volume recruitment in trials by helping the inclusion of patients unable to travel or with mobility issues, improving participant representativeness, and reducing selection bias. Participants’ engagement should also be increased and limit attrition in the studies by involving them in the management of their pathology.

Integrating vocal biomarkers in care would also facilitate communication between patients and medical teams thanks to better follow-up and medical care; particularly in a pandemic, it would limit contacts and infection risks. Coupling vocal biomarkers with alert systems could improve patient care and safety. The inclusion of voice analysis in health calls or emergency centers would enable augmented consultations, more accurate caller authentication, and real-time analysis of important health-related features [21].

We believe that increased use of voice in the future will maximize the benefits for both the investigators and the study participants, thanks to the combination of the best of both traditional and digital approaches; it will ultimately increase the quality of the studies and saves time and costs. Besides, both for clinical practice and clinical research, there is a need to avoid the ‘in-between clinical visits black hole’ and to describe better what is happening to a patient between two follow-up visits. Telemonitoring solutions based on vocal biomarker monitoring could allow for a more accurate follow-up and complement on-site evaluations [28].

However, some researchers have challenged the relevance of a vocal biomarker for COVID-19 detection [29] and raised the issue of whether it is an actual marker of the disease or a proxy of the general health status, or worse, a proxy of the context of recording of the individuals.

Other limitations to the use of vocal biomarkers for remote monitoring of COVID-19–related symptoms include patients’ acceptability and readiness of the health care system [19] for this new technology. The health status could also be a limitation, in the way that persons experiencing severe symptoms could be too affected to be willing to do the voice recording regularly, and therefore, affect adherence to the digital solution.

## Development of a Digital Health Solution Based on Vocal Biomarkers to Monitor COVID-19–Related Symptoms

### Identification of the Outcome to Be Monitored

Many previously cited COVID-19–related symptoms could theoretically be monitored using voice, including fatigue, dyspnea, loss of taste or smell, disease severity, presence or absence of symptoms, as well as impact on mental health (eg, stress, anxiety, and depression). However, the choice of monitoring one of these symptoms should be discussed with health care professionals to ensure its clinical relevance.

**Figure 1:**
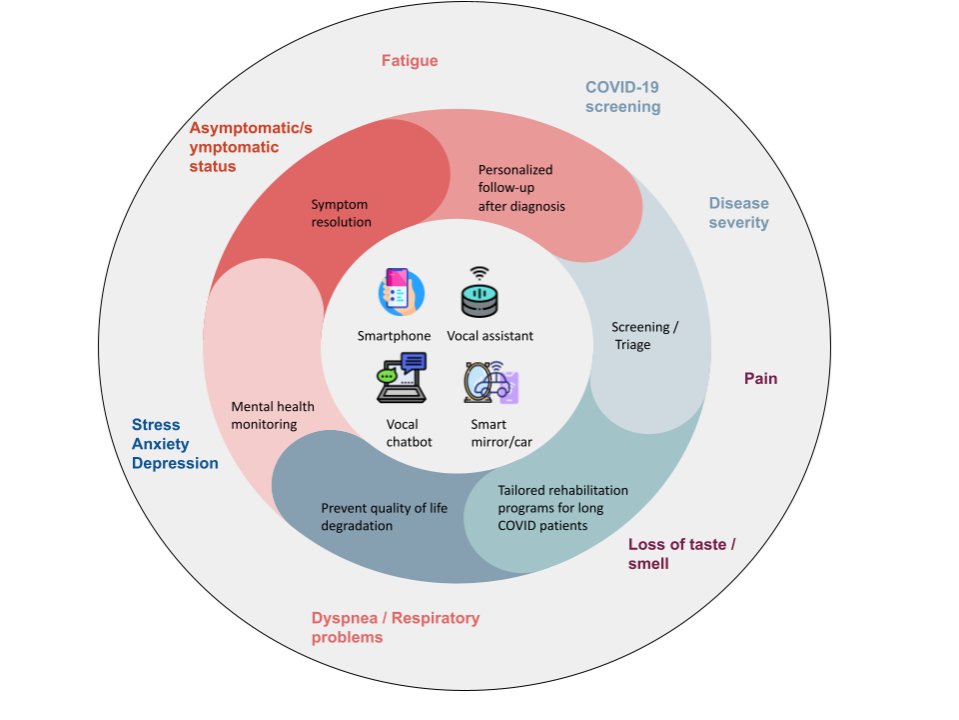
Different use case of vocal biomarkers related to COVID-19. The outside layer represents different vocal biomarkers , the second layer represents the use cases, and the center shows different devices that could embark vocal biomarkers.

Potential clinical applications of vocal biomarkers for these symptoms are presented in Figure 1. Vocal biomarkers could be implemented in devices, such as smartphone apps, chatbots, smart mirrors or cars, and vocal assistants, to monitor symptom resolution (with a vocal biomarkers of symptomatic or asymptomatic status), mental health, and quality of life degradation (with vocal biomarkers of stress, anxiety, depression, chronic fatigue, or dyspnea); to propose personalized follow-up after diagnosis (with vocal biomarkers of disease severity); to perform a screening and a triage of patients at hospital (with vocal biomarkers of disease severity, pain, or loss of taste and smell); and to propose tailored rehabilitation programs for patients with long COVID (using vocal biomarkers of loss of taste and smell, pain, dyspnea, and fatigue).

### Data Collection

This step consists of voice data collection coupled with well-documented clinical data in screening platforms such as Colive Voice [30] or large prospective cohort studies [2,31]. The collected data have to be diverse enough and should represent the target population in terms of languages, accents, and socioeconomic backgrounds to decrease the risk of systemic biases and the risk of increasing a potential preexisting digital and socioeconomic divide in the population.

Different types of voice records, such as vowel phonation, reading a predefined text, counting, or semispontaneous voice tasks, as well as nonverbal vocalization (eg, coughing and breathing) can be collected depending on the future foreseen clinical application. The environment and conditions of voice recordings are critical, in particular in COVID-19 settings. Indeed, background noises and audio features may differ between COVID-19–positive and control participants due to the isolation of COVID-19–positive persons. For this reason, clear instructions to perform the voice recordings have to be provided before data collection.

Depending on the final use case, at home or at the hospital, recordings can be performed under either controlled conditions with high-quality microphones and standard processes or in real-life conditions with the patients’ smartphones. As mentioned before, the recording situation may impact audio quality but can also allow for the training of more relevant algorithms based on more diverse data sets. Wearing a surgical mask has been shown to have no impact on vocal parameters, such as vocal intensity, jitter, shimmer, and harmonics-to-noise ratio [32]; voice collection can thus be performed safely at the hospital or during clinical visits. The validity of the data set can also be a concern, as the COVID-19 status may be self-reported [33] and may induce some mislabeling of the audio.

### From Voice Recording Toward Vocal Biomarkers

After several preprocessing steps on the raw audio signals, the identification of vocal biomarkers candidates is based on machine and deep learning methods, such as support machine vectors, random forest, and visual geometry group, among many others, which can be supervised or unsupervised. When a limited data set is available, an alternative is to use transfer-learning methods. The interest of this method is to take advantage of a pretraining of the algorithm on a large data set from another domain and to fine-tune it for the defined target. Internal and external validations are then required in other settings and using other data sets.

At this stage, the vocal biomarker candidate has also to be clinically validated in one or several clinical studies in comparison to the gold standard measurement of the outcome of interest (eg, validated scales to assess fatigue or stress or, if available, established physiological parameters such as blood pressure or glycemia). The design of the studies has to be chosen carefully, going from a very standardized double-blind study to a real-life prospective study.

Recommendations for the 3 steps for the evaluation of vocal biomarkers (ie, verification of audio quality, analytical validation, and clinical validation) have been provided by Robin et al [34].

### Embedding the Vocal Biomarker in a Digital Health Solution

The next step after identification and validation of a promising vocal biomarker candidate is to design a digital health solution to embed it. Several digital devices can be imagined, such as smartphone apps, chatbots, smart mirrors, or voice assistants. The future end users of the device, namely the patients and the health care professionals, should be involved in the co-design of the final solution [35,36] to ensure it meets their needs and expectations. This is particularly important for voice-based technologies to ensure their future acceptability. Finally, feedback loops should be implemented to improve both the solution and the algorithm through lessons learned in population studies.

Particular caution should be taken when collecting voice; indeed, voice is considered as identifying and sensitive data, and its collection falls under different regulations or laws, such as General Data Protection Regulations [37] in Europe and Personal Information Protection and Electronic Documents Act in Canada. In the United States, there is no single data protection law but rather multiple laws enacted at a federal or state level. These different laws do not protect individuals at the same level, and to minimize future risks for the use of the digital health solution, it is highly recommended to obtain explicit consent prior to collection. Measures have to be implemented by design and by default to securely process voice without privacy leakage (eg, encryption of voice data, splitting data into random components, or using data representations from which sensitive identifiable information is removed).

Once the digital health solution is developed, an additional validation step is mandatory to prove clinical benefit, effectiveness, and security in clinical trials. Indeed, most digital health solutions fall under the new Medical Device Regulation [38]. CE marking or Food and Drug Administration (FDA) certification will be mandatory to bring the solution to the market, and requirements for clinical evaluation are diverse, depending on the final device. The definition of the ‘intend-to-use’ (article 2 of Medical Device Regulation) of the device should be done early in the development process to define clinical evaluation requirements. It is highly recommended to consult guidance documents for digital health interventions (eg, MEDDEV 2.1 [39], FDA’s benefit-risk framework for medical devices [40], and the World Health Organization’s monitoring and evaluating digital health interventions [41]) and to take advice from regulatory authorities or a notified body.

Requests for reimbursement of the device or solution can be made to national health insurance funds after the clinical and economic interest of the new digital system is proved.

**Figure 2:**
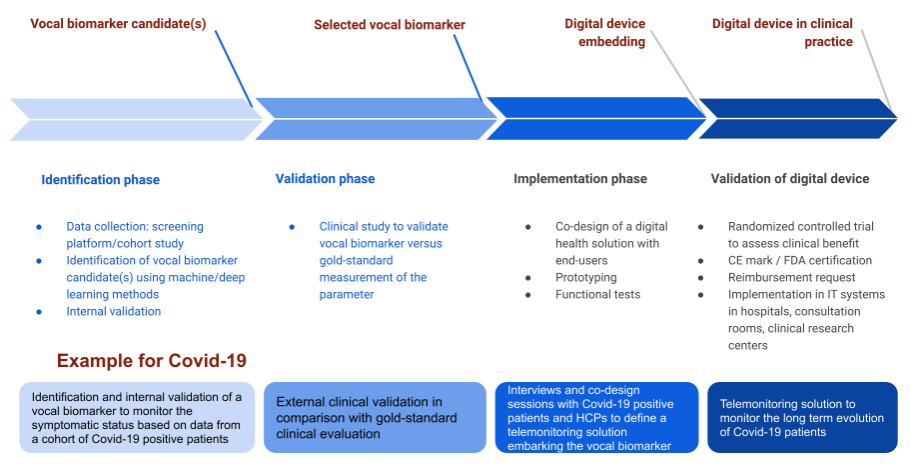
Pipeline from identification to implementation in clinical practice of a vocal biomarker. CE: conformite europeenne; FDA: Food and Drug Administration; HCP: health care provider; IT: information technology.

An overview of the pipeline to develop a digital remote monitoring solution based on vocal biomarkers is presented in Figure 2.

Finally, as mentioned above, some vocal biomarkers have been identified in several pathologies, but none of them are currently used in clinical or real-life practice; indeed, the field of vocal biomarkers is recent, and the way is still long until a health solution based on them can be commercialized. Companies are currently in the process of requesting FDA authorization or CE mark but are facing challenges related to data security, ethical issues, as well as reliability and reproducibility of the algorithms.

## Conclusions

This viewpoint presents the need for new digital remote monitoring technologies in the context of COVID-19 and the potential benefit of using vocal biomarkers for this purpose. We also propose a pathway and recommendations for a successful implementation in clinical practice of a digital health solution based on vocal biomarkers.

Implementation of vocal biomarkers in a digital solution for remote patient monitoring of frequently reported symptoms of COVID-19 is of high interest. Its full potential can be achieved in the short term but still includes challenging steps and hurdles to overcome before launching reliable solutions in practice. Vocal biomarker acceptability remains to be properly evaluated, as the use of voice is a rather new technique and needs to be integrated into existing health information technology systems. The future of digital solutions embedding such vocal biomarkers will be diverse and will probably evolve toward multitechnologies solutions combining voice, video, and sensors to offer the most comprehensive view of a patient’s health status.

## Abbreviations

FDA: Food and Drug Administration
